# 7-dehydrocholesterol efficiently supports Ret signaling in a mouse model of Smith-Opitz-Lemli syndrome

**DOI:** 10.1038/srep28534

**Published:** 2016-06-23

**Authors:** Myriam Gou-Fàbregas, Anna Macià, Carlos Anerillas, Marta Vaquero, Mariona Jové, Sanjay Jain, Joan Ribera, Mario Encinas

**Affiliations:** 1 Department of Experimental Medicine, Universitat de Lleida/Institut de Recerca Biomèdica de Lleida (IRB Lleida), Spain; 2 Metabolomics Service, IRB Lleida, Rovira Roure, 80, Lleida 25198, Spain; 3 Departments of Internal Medicine (Renal Division) and Pathology, Washington University School of Medicine, St. Louis, MO 63110, USA

## Abstract

Smith-Lemli-Opitz syndrome (SLOS) is a rare disorder of cholesterol synthesis. Affected individuals exhibit growth failure, intellectual disability and a broad spectrum of developmental malformations. Among them, renal agenesis or hypoplasia, decreased innervation of the gut, and ptosis are consistent with impaired Ret signaling. Ret is a receptor tyrosine kinase that achieves full activity when recruited to lipid rafts. Mice mutant for *Ret* are born with no kidneys and enteric neurons, and display sympathetic nervous system defects causing ptosis. Since cholesterol is a critical component of lipid rafts, here we tested the hypothesis of whether the cause of the above malformations found in SLOS is defective Ret signaling owing to improper lipid raft composition or function. No defects consistent with decreased Ret signaling were found in newborn *Dhcr7*^−/−^ mice, or in *Dhcr7*^−/−^ mice lacking one copy of *Ret*. Although kidneys from *Dhcr7*^−/−^ mice showed a mild branching defect *in vitro*, GDNF was able to support survival and downstream signaling of sympathetic neurons. Consistently, GFRα1 correctly partitioned to lipid rafts in brain tissue. Finally, replacement experiments demonstrated that 7-DHC efficiently supports Ret signaling *in vitro*. Taken together, our findings do not support a role of Ret signaling in the pathogenesis of SLOS.

Smith–Lemli–Opitz syndrome (SLOS; OMIM 270400, ORPHA818) is an autosomal recessive, multiple congenital malformation syndrome, with an incidence of approximately 01/50,000 live births. SLOS presents with a very heterogeneous phenotype, causing perinatal death in its most severe forms. In general, children affected by SLOS show growth retardation, developmental delay and intellectual disability. Craniofacial defects include microcephaly, micrognathia, and ptosis (dropping of the eyelid). Individuals affected by SLOS often display postaxial polydactyly of the hands or feet, and syndactyly of the 2nd and 3rd toes. Internal malformations include renal hypoplasia, ambiguous genitalia in male, pyloric stenosis, colonic aganglionosis (Hirschsprung disease), holoprosencephaly and cardiac malformations. SLOS patients are hyperactive, display self-injurious behavior, and demonstrate autistic characteristics [reviewed in^1^,^2^,^3^].

SLOS is caused by inherited mutations of the 7-dehydrocholesterol reductase gene (*DHCR7*)^4^,^5^. *DHCR7* gene product catalyzes the last step of cholesterol biosynthesis, namely conversion of 7-dehydrocholesterol (7-DHC) into cholesterol. As a consequence, *DHCR7* deficiency impairs cholesterol production, resulting in elevated 7-DHC levels and typically decreased cholesterol levels. Cholesterol is a major structural component of plasma membranes, is the precursor of steroid hormones, bile acids and constitutes a critical component of plasma membrane microdomains known as lipid rafts. Moreover, cholesterol is necessary for proper function of the sonic hedgehog (Shh) pathway^6^, and 7-DHC is the precursor of vitamin D. Given the multiple and important functions of cholesterol, it is not surprising that the molecular mechanisms by which such altered cholesterol/7-DHC ratio cause the variety of developmental abnormalities observed in SLOS patients are only partially understood.

The GDNF family ligands (GFLs) constitute a group of neurotrophic factors (GDNF, NRTN, PSPN, ARTN) that regulate several aspects of the developing nervous and genito-urinary systems^7^. GFLs signal through a common tyrosine kinase receptor (Ret), and one of the four co-receptors known as GFRα, which bind directly to GFLs providing ligand specificity. GFRα co-receptors are attached to the outer leaflet of the plasma membrane via a glycosyl-phosphatidyl inositol anchor, which targets them to lipid rafts. Stimulation by GFLs prompts recruitment of Ret to lipid rafts, where the GFL-GFRα-Ret ternary complex interacts with Src family kinases to elicit maximal biological responses^8^. Interestingly, stimulation of Ret by soluble GFRα1 also drives Ret to lipid rafts where it interacts with FRS2^9^. Moreover, partitioning of Ret to lipid rafts not only promotes association with key signaling intermediates, but prevents its proteasomal-mediated degradation^10^. Finally, a very recent report shows that knockin mice expressing a GFRα1 mutant localized outside lipid rafts phenocopy several aspects of Ret knockout mice, confirming the importance of GDNF-driven translocation of Ret to lipid rafts for its proper function^11^.

*In vivo*, Ret signaling is necessary for the proper development of the autonomic nervous system and the genito-urinary system. Mice bearing a variety of *Ret* mutations (as well as mutations in certain GFLs or their co-receptors) show different degrees of colonic aganglionosis, thus mimicking Hirschsprung disease in humans. On the other hand Ret signaling is crucial to kidney development, and mice bearing mutations in *Ret* or its co-receptor *GFRα1* develop several kidney defects ranging from complete agenesis to different degrees of kidney hypoplasia^12^,^13^,^14^,^15^,^16^,^17^,^18^. Finally, mice lacking either *ARTN* or *GFRα3* develop ptosis owing to failure of sympathetic neurons form the cervical superior ganglion to innervate the tarsus muscle^19^,^20^.

Given the overlapping spectra of congenital abnormalities found both in SLOS patients and *Ret* mutant mice and the necessity of intact lipid rafts for optimal Ret function, in the present work we aimed to elucidate whether abnormal Ret signaling plays a role in the pathogenesis of SLOS. Analysis of the genito-urinary and enteric nervous systems of *Dhcr7*^−/−^ or *Dhcr7*^−/−^*; Ret*^+*/EGFP*^ mice revealed no abnormalities consistent with compromised Ret signaling. Although kidneys from mutant mice branched at a slower pace than those from wild type littermates when explanted in the absence of exogenous cholesterol, sympathetic neurons from mutant mice showed normal responses to GDNF under the same conditions. Moreover, critical components of Ret signaling including GFRα1 or Src family kinases from knockout mice partitioned to the lipid raft fraction in flotation gradients, suggesting that 7-DHC allows formation of lipid rafts that efficiently support Ret signaling. In support with this notion, removal of cholesterol followed by replenishment with 7-DHC efficiently supported GDNF-mediated phosphorylation of Akt in MG87 fibroblasts cultured in the absence of cholesterol. Collectively, the data indicate that aberrant Ret signaling is unlikely to play a major role in the pathogenesis of SLOS.

## Results

### Urinary and enteric nervous systems are largely normal in newborn *Dhcr7* knockout mice

**Figure 1:**
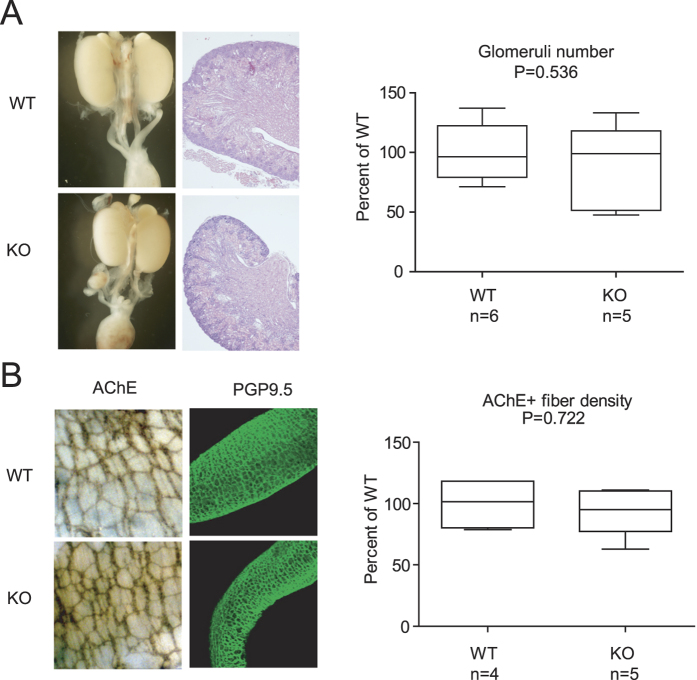
Genito-urinary and enteric nervous systems do not show developmental alterations in a SLOS mouse model. (**A**) Macroscopic and microscopic appearance of kidney and lower urinary tracts (left panel), and glomeruli number (right panel) of newborn wild type and *Dhcr7* knockout mice. (**B**) Representative images of acetyl cholinesterase (AChE) and PGP9.5 staining (left panel) and acetyl cholinesterase-positive fiber density (right panel) of newborn wild type and *Dhcr7* knockout mice.

Although no major defects in kidney and digestive system were described in *Dhcr7* knockout mice, we sought to investigate whether subtle abnormalities reflecting compromised Ret signaling could be identified in these mice. Macroscopic examination of the genito-urinary system of newborn mutant mice revealed no overt malformations consistent with aberrant Ret signaling such as kidney aplasia or hypoplasia, cystic or supernumerary kidneys, or ureter abnormalities (Fig. 1A, left panel). Hematoxylin-eosin staining showed that mutant kidneys had normal architecture, with well-organized cortex and medulla, a prominent nephrogenic zone, and no cystic dilations or signs of hydronephrosis (Fig. 1A, left panel). The number of glomeruli was also similar between *Dhcr7* knockout mice and their wild type littermates (Fig. 1B), consistent with proper branching of the ureteric bud. We next wanted to check the status of enteric nervous system. Immunostaining of colon from newborn mice with PGP9.5, which labels enteric neuronal precursors^21^ showed no signs of colonic aganglionosis, the hallmark of Hirschsprung disease (Fig. 1B, left panel). Enteric plexus density was assessed throughout the entire gut by means of acetylcholinesterase staining, which labels cholinergic enteric neurons, revealing no differences between genotypes (Fig. 1B, right panel).

### Removal of one *Ret* allele does not cause renal or enteric abnormalities in *Dhcr7* knockout mice

**Figure 2:**
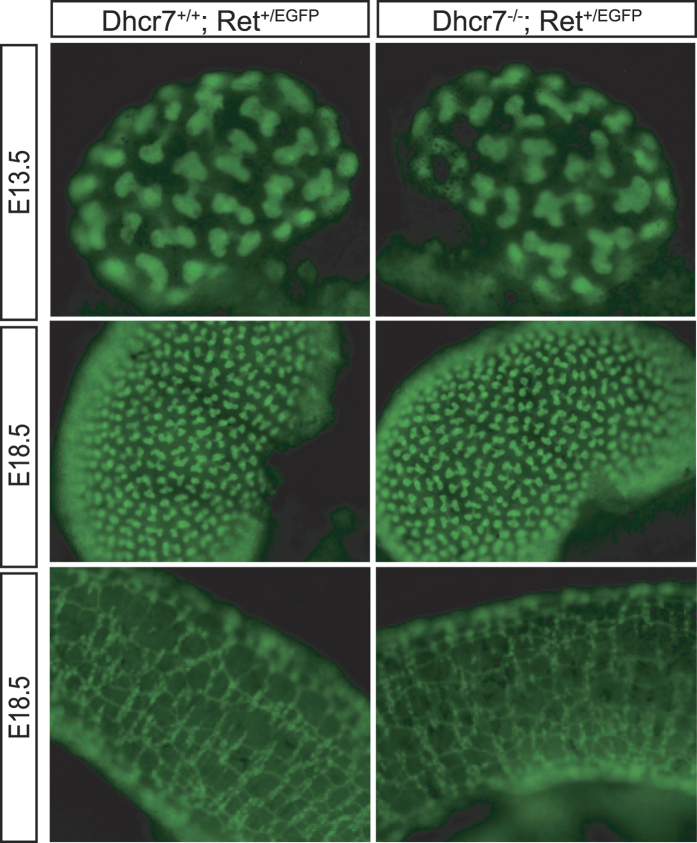
Reducing *Ret* dosage does not unveil developmental defects of the genito-urinary and enteric nervous systems of *Dhcr7* knockout mice. Representative fluorescence images metanephroi (top and middle panel) and colon (bottom panel) from *Dhcr7*^+*/*+−^; *Ret*^+*/EGFP*^ and *Dhcr7*^−/−^*; Ret*^+*/EGFP*^ embryos of the indicated ages. No overt differences in ureteric bud branches or plexus density are observed.

The above results were not surprising in light of the relatively mild phenotype of *Dhcr7* null mutants when compared to SLOS patients. Besides cleft palate and immature lungs, *Dhcr7* knockout mice show no overt developmental abnormalities reminiscent of the human disease^22^. It is usually assumed that such discrepancies are due to differences in transplacental cholesterol transport between species, being it obligated in rodents^23^,^24^ but not humans^25^. Thus, maternal cholesterol transfer to embryos, possibly via Abca1 cholesterol transporter^26^, would compensate cholesterol deficiency in murine but not human embryos. To determine whether reduction in *Ret* gene dosage could unveil suboptimal Ret signaling in the context of *Dhcr7* deficiency, we crossed *Dhcr7* mutant mice to heterozygous *Ret* knockin mice expressing one copy of *EGFP* from the *Ret* locus^27^, thus allowing easy tracking of Ret-expressing cells. As shown in Fig. 2, removal of one copy of *Ret* did not affect ureteric bud branching or enteric plexus density (n ≥ 3 for each genotype), thus reinforcing the idea that no major abnormalities related to abnormal Ret signaling are found in *Dhcr7* knockout mice. Taken together, the above observations are not consistent with abnormal Ret function in the developing urinary and nervous systems of *Dhcr7* knockout mice.

### Ureteric bud branching of *Dhcr7* mutant metanephroi is reduced *in vitro*

**Figure 3:**
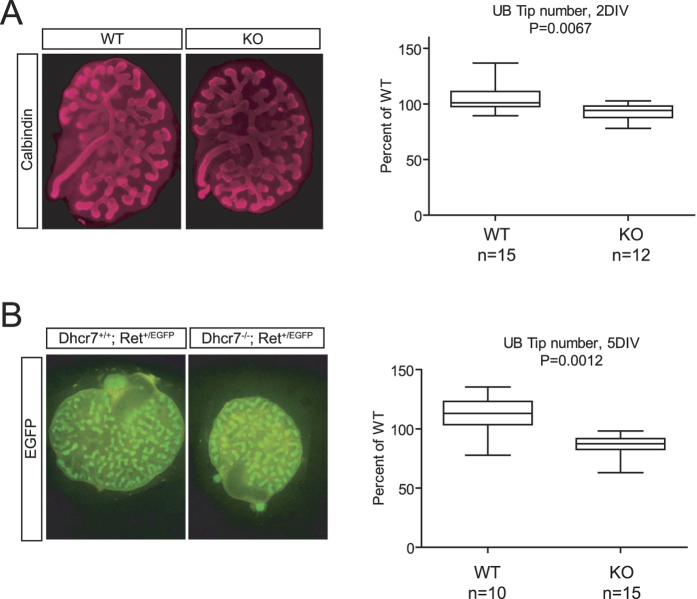
Ureteric bud *in vitro* branching is modestly reduced in *Dhcr7* knockout mice. (**A**) Calbindin immunostaining of E12.5 kidney explants of the indicated genotypes cultured in the absence of exogenous source of cholesterol for 2 days (left panel). Quantification of ureteric bud tip number after 2 days *in vitro* (2DIV). (**B**) EGFP immunofluorescence of E12.5 kidney explants of the indicated genotypes cultured in the absence of exogenous source of cholesterol for 5 days (left panel). Quantification of ureteric bud tip number after 5 days *in vitro* (5DIV) (right panel).

To examine Ret signaling avoiding the confounding effect of maternal cholesterol transfer, we decided to place embryonic metanephroi from *Dhcr7* mutant embryos in culture, in the absence of any exogenous source of cholesterol. As stated in the introduction, ureteric bud branching is critically dependent on GDNF-Ret signaling, both *in vivo* and in explant culture. We explanted metanephroi from E12.5 wild type and null embryos in defined medium containing retinoic acid, and counted ureteric bud tips after two and five days *in vitro*. We pooled data from wild type and *Dhcr7* knockout mice irrespectively of the *Ret* allele expressed as no differences on ureteric bud branching were found between *Ret*^+*/EGFP*^ and *Ret*^+*/*+^ embryos within a given *Dhcr7* genotype (data not shown). We then stained *Ret*^+*/*+^ metanephric explants with an antibody against CalbindinD-28k, a marker of the ureteric bud^28^, or directly photographed *Ret*^+*/EGFP*^ metanephroi under a fluorescence microscope. We used both techiniques at both 2 days *in vitro* and 5 days *in vitro*. As shown in Fig. 3A, small but significant differences between wild type and *Dhcr7* knockout metanephroi were found after two days in culture, which increased with time in culture (Fig. 3B). Thus, ureteric bud branching is reduced in *Dhcr7* null embryonic kidneys when cultured in defined medium and are therefore devoid of exogenous cholesterol.

### Ret signaling is not affected by deletion of *Dhcr7* in sympathetic neurons

**Figure 4:**
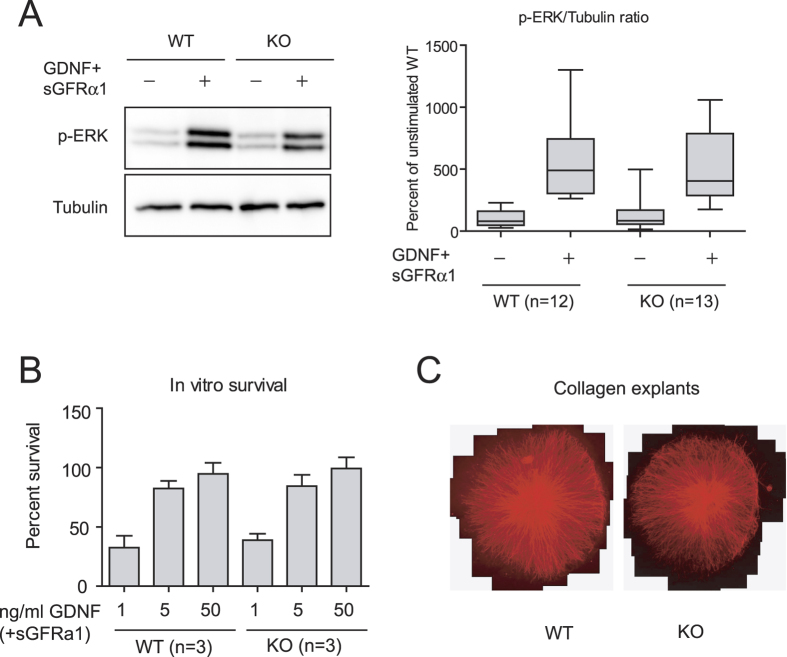
*Dhcr7*^−/−^ sympathetic neurons properly respond to GDNF stimulation. (**A**) Superior cervical ganglion neurons from wild type and *Dhcr7* knockout were cultured and stimulated with GDNF as described in methods. Total lysates were resolved by SDS-PAGE and probed with the indicated antibodies (left panel). The graph on the right panel shows the relative intensity of phospho-ERK signal normalized by tubulin. (**B**) GDNF-mediated survival of SCG neurons was evaluated after 48 hours in the presence of growing concentrations of GDNF. (**C**) Representative images of SCG explants after five days in the presence of 50 ng/ml GDNF.

Even though signaling by the GDNF-GFRα1-Ret axis is the major player in ureteric bud branching, the above observations do not directly prove suboptimal Ret signaling in this system. To directly assess biological responses downstream of Ret, we chose sympathetic neurons from the superior cervical ganglion as a model system. The importance of lipid rafts in Ret signaling has been extensively characterized in sympathetic neurons from the superior cervical ganglion (SCG)^10^. We first used immunoblots to phosphorylated ERK as a readout of Ret signaling in sympathetic neurons cultured for five days in the presence of lipid-depleted serum, and stimulated with GDNF in the presence soluble GFRα1. As shown in Fig. 4A, no major differences on GDNF-mediated ERK phosphorylation were found between wild type and mutant neurons. We next assessed survival of sympathetic neurons in the presence of increasing concentrations of GDNF and a fixed concentration of sGFRα1. As shown in Fig. 4B, no differences between genotypes were observed at any of the concentrations assayed. Neurite outgrowth of SCG explants in response to GDNF was also similar between wild type and *Dhcr7* null mice (Fig. 4C). Thus, at least for sympathetic neurons, mutation of *Dhcr7* does not appear to have any consequence on GDNF-induced Ret signaling.

### GFRα1 partitions to lipid rafts in brains from *Dhcr7* null mice

**Figure 5:**
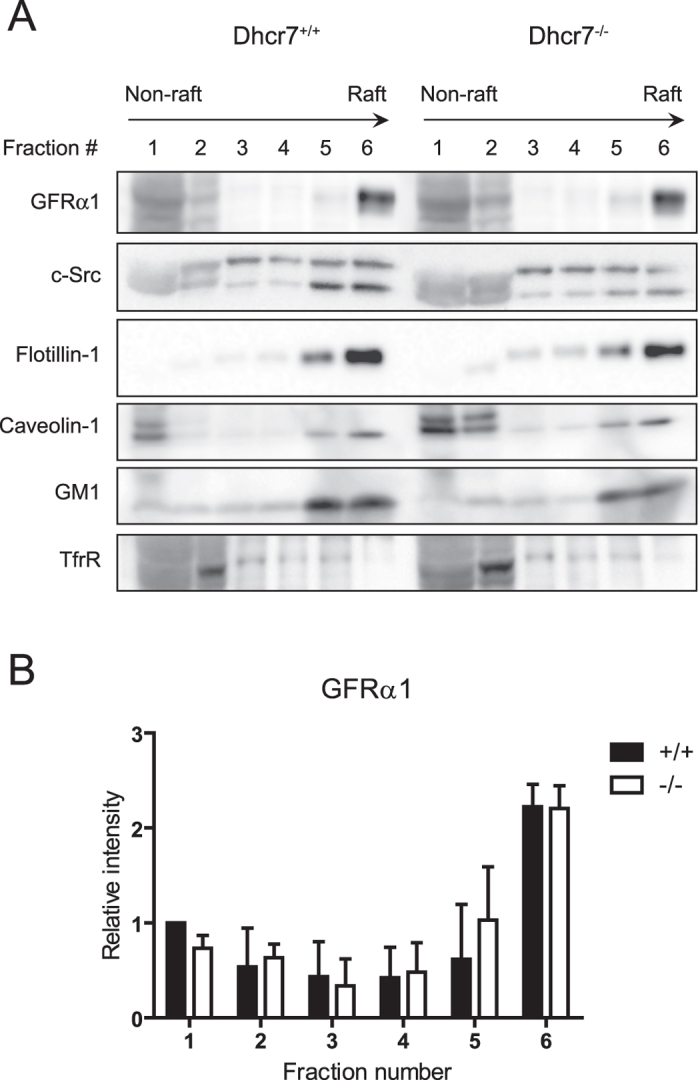
The Ret co-receptor GFRα1 is properly located to lipid raft fractions in *Dhcr7*^−/−^ mice. (**A**) Representative immunoblots of consecutive fractions from wild type and *Dhcr7* knockout brain homogenates subjected to sucrose density centrifugation. Six equal fractions were taken from the top of the tubes and labeled from fraction 1 (non-rafts) to fraction 6 (rafts). Protein in fractions was resolved by SDS-PAGE and probed with the indicated antibodies. (**B**) Relative intensity of GFRα1 bands (mean ± SEM) from three independent experiments.

**Figure 6:**
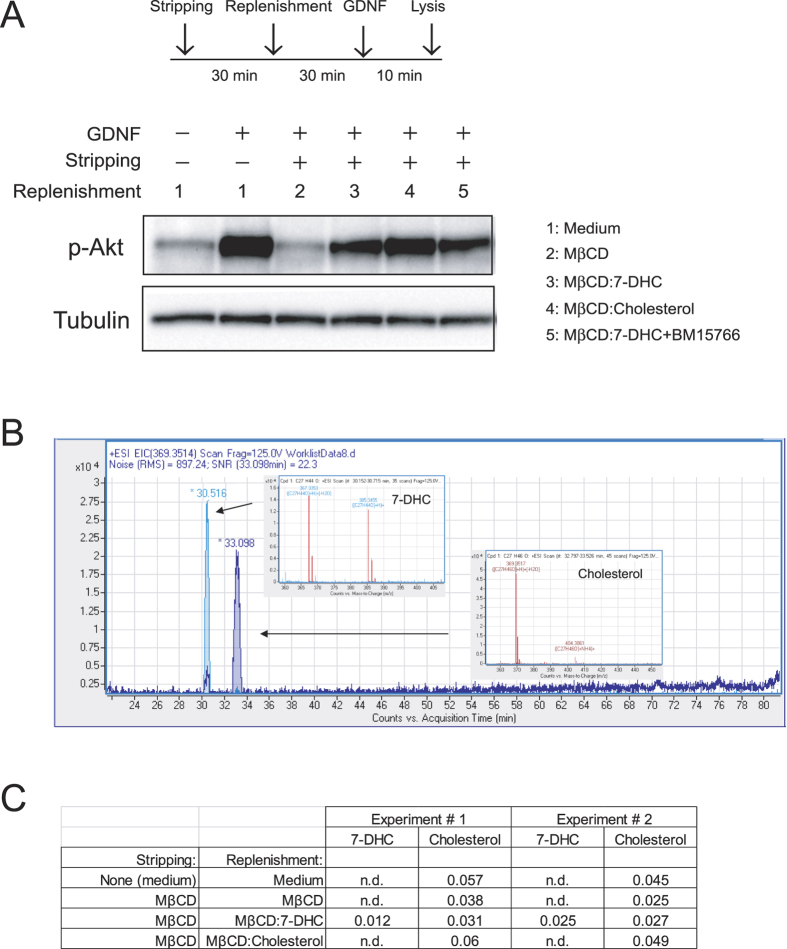
7-DHC supports Ret signaling. (**A**) MG87 cells were treated or not with MβCD for 30 min to deplete cholesterol (stripping), followed by 30 min replenishment step with MβCD alone, loaded with 7-DHC or with cholesterol, or with 7-DHC in the presence of the Dhcr7 inhibitor BM15766. Cells were then stimulated with GDNF for 10 minutes. Total lysates were resolved by SDS-PAGE and probed with the indicated antibodies. (**B**) Representative extracted ion chromatograms and MS spectrum for 7-DHC and Cholesterol in samples from cells replenished with MβCD:7-DHC complexes. Chromatograms shown retention times of the compounds and the MS peak used for quantification. The ions selected for quantification was m/z (M + H) + (−H2O): 367.3360 for 7-DHC and m/z (M + H) + (−H2O): 369.3519 for cholesterol. (**C**) Relative abundances of 7-DHC and cholesterol measured by means of LC-QTOF-MS in two independent experiments. Numbers indicate the ratio of the indicated metabolite (7-DHC or cholesterol) with respect to an internal standard (^13^C-cholesterol). N.d.: not detected.

As stated in the introduction, GFRα1, which is located to lipid rafts by virtue of its glycosylphosphatydilinositol anchor, recruits Ret to these membrane domains upon ligand stimulation. We next investigated whether lack of effect of *Dhcr7* mutation on Ret bioactivity in neurons was due to normal partition of GFRα1 to lipid rafts. To this end, we generated flotation gradients from neonatal brain extracts of both control and *Dhcr7* null mice. Importantly, cholesterol does not cross the blood-brain barrier and thus the contribution of maternal cholesterol to central nervous system drops from E10-11 onwards, being 90% of neonatal brain cholesterol of embryonic origin^29^. Therefore, 7-DHC but not cholesterol is the major sterol in brains from *Dhcr7* knockout mice. As shown in Fig. 5, GFRα1 was mainly found in the high-buoyant, lipid raft fractions, in both wild type and *Dhcr7* mull mice. Partition of other critical components of Ret signaling such as Src family kinases was also similar in wild type and *Dhcr7* knockout mice (Fig. 5). Distribution of lipid raft (GM1 ganglioside, caveolin-1 and flotillin-1) and non-raft (transferrin receptor) markers indicated correct fractionation of the samples. Thus, loss of *Dhcr7* does not affect distribution of critical components of Ret signaling to lipid rafts in brain, suggesting that 7-DHC can efficiently support Ret signaling. To directly test this hypothesis, we conducted cholesterol depletion followed by 7-DHC replenishment experiments. We first confirmed that depletion of membrane cholesterol by means of methyl-β-cyclodextrin (MβCD) affected signaling downstream of Ret in the mouse fibroblast cell line MG87 stably expressing Ret and GFRα1, a widely used model to study Ret signaling. As shown in Supplemental Fig. 1, GDNF-mediated phosphorylation of both ERKs and Akt were affected by increasing concentrations of MβCD to different extents, being the PI3-K/Akt pathway more sensitive to cholesterol depletion. Since MβCD was toxic at high concentrations even for short exposure times, we used the lowest effective concentration of this compound (4 mM) and focused on p-Akt phosphorylation for replenishment experiments. As shown in Fig. 6A, replenishment of cellular membranes with 7-DHC supported GDNF-mediated Akt phosphorylation to levels comparable to those obtained in parallel cultures not subjected to depletion/replenishment treatments, or to cells replenished with cholesterol. Interestingly, replenishment with 7-DHC in the presence of BM15766, a novel Dhcr7 inhibitor, did not substantially changed Akt phosphorylation, indicating that conversion of 7-DHC to cholesterol was not responsible for rescuing the pathway. To assess the efficacy of these stripping/replenishment experiments, we measured both 7-DHC and cholesterol in cell extracts by means of liquid chromatography quadrupole time-of-flight mass spectrometry (LC-QTOF-MS/MS), as described in methods. MβCD depleted approximately half of the cellular cholesterol, which was efficiently replenished by MβCD:Cholesterol. Importantly, MβCD:7-DHC complexes did not increase cholesterol above levels found in cells treated with MβCD alone, indicating that 7-DHC was not being converted to cholesterol in cells exposed to MβCD:7-DHC. Finally, we only detected 7DHC in cells treated with MβCD:7-DHC complexes but not in the rest of conditions (Fig. 6B,C). Taken together, these experiments indicate that 7-DHC efficiently supports Ret signaling.

## Discussion

In the present work we examined the hypothesis of whether suboptimal Ret signaling could explain some of the developmental abnormalities found in SLOS patients. Such hypothesis was formulated based on the overlapping phenotypes of SLOS patients and *Ret* mutant mice, and the observation that lipid rafts are critical for proper Ret signaling.

Even though the genetic defect causing SLOS is well-established, the molecular mechanisms leading to the plethora of developmental malformations found in SLOS patients are only partially understood. Besides being a fundamental component of plasma membranes, cholesterol serves as precursor for a number of important biomolecules such as bile acids, steroid hormones and signaling molecules. Such multiplicity of functions makes it unlikely that a single molecular mechanism underlies the pathogenesis of SLOS. Moreover, not only cholesterol deficiency but the toxic effects of 7-DHC (or its derived oxysterols) buildup may account for some of the complications found in SLOS patients^3^,^30^. A number of observations suggest that membrane structural and functional properties may be affected in SLOS patients. 7-DHC has been shown to induce changes in the physico-chemical properties of both artificial and cellular membranes, including fluidity, electric potential, and curvature^31^,^32^,^33^,^34^. These changes likely account for functional alterations of membrane proteins such as the NMDA and serotonin receptors^35^,^36^. Of particular interest are the abnormalities found in lipid rafts, as cholesterol is one important component of these membrane microdomains. Thus, 7-DHC has been shown to induce changes in both the lipid and protein composition of rafts^37^,^38^. Likewise, changes in lipid raft stability may account for impaired degranulation responses of mastocytes in response to IgE^39^, or for alterations in caveolae and the potassium channel BK_Ca_ in skin fibroblasts^40^. Despite these observations, our data show that GFRα1 (and Src-family kinases) equally partition to lipid raft fractions in brain extracts from both wild type and Dhcr7 null mice, suggesting that at least in central neurons Ret would be correctly recruited to lipid rafts upon GDNF stimulation. It is important to stress that brain cholesterol is synthesized by the embryo from E11.5 onwards, and therefore maternal cholesterol is likely to be residual in brains from newborn *Dhcr7* null mice. Even though GFRα1-mediated recruitment is not the only mechanism by which Ret moves to lipid rafts upon ligand stimulation^9^, recent *in vivo* data show that it constitutes the major mechanism involved in such translocation^11^.

We have found that embryonic kidney explants from knockout mice branch at a slightly slower pace than their wild type counterparts. Even though GDNF-Ret signaling is the master regulator of kidney branching, we do not know whether these defects are caused by impaired Ret signaling in mutant kidneys. In light of our results with sympathetic neurons we find such possibility unlikely, although we cannot exclude the possibility that sensitivity of Ret signaling to 7-DHC varies depending on the cellular context. Thus, mice expressing a ‘non-raft’ version of *GFRα1* (*GFRα1*^*TM/TM*^ mice) show defects on urogenital, enteric and motor but not sympathetic nervous system^11^. However, the renal phenotype of *GFRα1*^*TM/TM*^ mice is a fully penetrant, complete kidney agenesis, whereas we observe a modest branching defect that at the most suggests that 7-DHC partially supports Ret signaling in the ureteric bud. Alternatively, other signaling molecules that play a secondary role in kidney branching might be affected by lack of cholesterol/increased levels of 7-DHC. Thus, signaling by FGF10 is able to induce kidney morphogenesis in animals lacking *Ret* and *Sprouty1*^41^. Similarly, perturbation of Shh signaling causes kidney defects in mice ranging from kidney hypoplasia to kidney agenesis^42^,^43^. Interestingly, altered SHH signaling is consistent with some SLOS features such as holoprosencephaly and polydactyly, and previous cell culture work demonstrates diminished responses to Shh in cholesterol-depleted cells^44^. However, the effect of Dhcr7 on the activity of the Shh pathway is controversial. Studies in Xenopus show that Dhcr7 negatively regulates the pathway by inhibiting Smo, in a reductase-independent manner^45^. In contrast, Bijlsma and colleagues^46^ show that secretion of pro-vitamin D3 (whose precursor is 7-DHC) inhibits Smo activity, suggesting that Dhcr7 should activate the pathway by keeping the levels of 7-DHC low. In contrast, a more recent work provides compelling genetic and pharmacological evidence that reduced cholesterol rather than increased 7-DHC or oxysterol levels block Shh pathway by impairing Smo activity^47^. All these observations point to a complex regulation of the Shh pathway by sterols beyond their role as covalent modifications of the Shh molecule. Another intriguing mechanism by which 7-DHC might regulate kidney morphogenesis is through modulation of the Wnt/β-catenin pathway. Thus, a very recent report establishes that elevated 7-DHC but not diminished cholesterol levels are responsible for aberrant neuronal differentiation of induced pluripotent stem cells derived from SLOS patients, via inhibition of the Wnt/β-catenin pathway^48^. Interestingly, genetic inactivation of β-catenin in the ureteric bud in mice leads to kidney agenesis/dysgenesis owing to reduced ureteric bud branching^49^.

In conclusion, our results show that 7-DHC has the ability to support Ret signaling in several model systems, indicating that impaired Ret function is unlikely to contribute to the pathogenesis of SLOS.

## Methods

### Mice

All experimental procedures involving mice were in accordance with national and regional guidelines and approved by the experimental Animal Ethic Committee of the University of Lleida. *Dhcr7* null mice^22^ were purchased from Jackson Laboratories (Bar Harbor, Maine). Mice bearing a floxed Ret allele followed by EGFP (*Ret*^*floxEGFP*^) have been previously described^27^. These mice were crossed to transgenic mice expressing Cre recombinase under the control of phosphoglycerate kinase 1 promoter [Jackson Laboratories^50^] leading to widespread excision of the floxed *Ret* allele and subsequent expression of *EGFP* from the *Ret* locus.

### Acetylcholinesterase staining

Newborn mouse gut was dissected and fixed in 4% paraformaldehyde for 1 to 2 h at 4 °C, transferred to saturated sodium sulfate, and stored overnight at 4 °C. Acetylcholinesterase staining was then performed exactly as described^51^. Quantification of enteric plexus fiber density was determined from randomly selected fields per mouse, by counting the number of upper and right side crossing fibers points of a defined grid as described by^52^.

### Nephron number assessment

Kidneys from wild type and *Dhcr7* knockout newborn littermates were dissected, fixed in 4% paraformaldehyde, dehydrated and embedded in paraffin blocs. Histological analysis was performed on serial 5 μm sections stained with Hematoxylin and Eosin. Kidneys were sectioned and glomeruli were counted as previously described^53^.

### Cell culture

Neurons from the sympathetic superior cervical ganglion of newborn *Dhcr7* mice cultured as described^54^ with minor modifications. Briefly, ganglia were enzymatically digested, dissociated and plated on rat tail collagen-coated dishes in medium containing 50 ng/ml NGF (Alomone labs, Jerusalem, Israel) and 10% lipid-depleted fetal bovine serum (Life Technologies, Carlsbad, CA) for 5–7 days. For survival experiments cells were incubated with the indicated concentrations of GDNF plus 100 ng/ml Fc-GFRα1 (R & D Systems, Minneapolis, MN). Neuronal survival was assessed by counting neurons of designated fields before and after treatments at the indicated time points. For immunoblots cells were treated in complete medium as above, deprived of NGF overnight, and stimulated for 10 minutes with 50 ng/ml GDNF plus 100 ng/ml Fc-GFRα1. For SCG explants, whole superior cervical ganglion from newborn Dhcr7 mice were dissected and plated in collagen matrices in the presence of NGF and charcoal stripped serum (Life Technologies, Carlsbad, CA). Twenty four hours later, each well was washed three times with PBS and switched to medium supplemented with GDNF (50 ng/ml) and charcoal stripped serum for five days. MG87 fibroblasts stably expressing Ret and GFRα1 were maintained in DMEM plus 10% lipid-depleted FBS.

### Metanephric kidney explants

Metanephroi were dissected from E12.5 embryos, placed on 0.4 μm pore PTFE inserts (Millipore), and cultured on air-fluid interphase with defined media in the bottom chamber [DMEM, 1x ITS supplement (Life Technologies) and 200 nM all-trans retinoic acid (Sigma, St. Louis, MO)]. Microscopy images were obtained at the indicated time points, and the number of ureteric buds was counted and expressed as percent of wild type. Culture media was changed every 48 h.

### Flotation gradients

A slight modification of the method used by Tansey and collaborators^8^ was used. Brains from newborn mice were homogenized in 0.5 ml of 0.1% Triton lysis buffer [50 mM Tris-HCl (pH 7.4), 150 mM NaCl, 5 mM EDTA, 0.1% Triton X-100, 10 mM NaF, 1 mM Na2VO4, 50 mM β-glycerophosphate, 2 mM Benzamidine, 1 μg/ml leupeptin and 1 μg/ml aprotinin] using a dounce tissue grinder, followed by four passages through a 22G needle. Homogenates were centrifuged at 3000 rpm at 4 °C for 10 min, and supernatants were adjusted to 35% sucrose, placed in a SW55Ti ultracentrifuge tube (Beckman), and overlayed with 3.5 ml of 30% Sucrose in 0.1% Triton lysis buffer and 0.5 ml of 0.1% Triton lysis buffer. After centrifugation (4 h, 200,000 × g, 4 °C), six fractions were collected from the top, TCA-acetone precipitated, air dried, and resuspended in 50 μl of Laemli sample buffer.

### Western blot

Western blot was conducted as previously described^54^ using primary antibodies listed in Supplementary Table 1. Immunoblots were developed using a VersaDoc device (BioRad, Hercules, CA), and densitometry performed using the software package ImageLab (BioRad). Ganglioside GM1 was visualized by directly probing membranes with HRP-conjugated cholera toxin B subunit (Millipore).

### Whole-mount immunofluorescence

Tissues were fixed in 4% paraformaldehyde and incubated in blocking buffer (PBS containing 0.2% Triton X-100 and 2% BSA) overnight at 4 °C. Tissues were then incubated with the indicated primary antibodies (Supplementary Table 1) in blocking buffer overnight at 4 °C, washed several times in blocking buffer at room temperature, and incubated with the appropriate DyLight-labeled secondary antibodies (Jackson ImmunoResearch Europe, Newmarket, Suffolk, UK) for 1 h at room temperature (metanephric explants) or overnight at 4 °C (guts).

### Manipulation of membrane cholesterol

Membrane cholesterol was depleted by adding 4 mM MβCD (Sigma) dissolved in serum-free medium to cells for 30 min at 37 °C. For replenishment experiments, cholesterol depletion with 4mM MβCD was followed by a 30 min incubation with 4 mM MβCD alone, or complexed with 0.4 mM 7-DHC or 0.4 mM cholesterol in serum free medium. 7-DHC was complexed to MβCD following the method of Christian and collaborators^55^. Briefly, 7-DHC was dissolved in chloroform:methanol 1:1 (v:v) to a concentration of 50 mg/ml. An aliquot of this solution was added to a glass tube and evaporated. Then the appropriate volume of MβCD dissolved in serum-free medium was added, sonicated and mixed overnight at 37 °C. Solutions were filtered through a 0.45 μM filter before use. Cholesterol: MβCD complexes were purchased from Sigma and adjusted to 1:10 molar ratio with MβCD. When indicated, BM15766 (Sigma) was included at a final concentration of 5 μM. Cells were then washed three times and stimulated with or without GDNF (50 ng/ml) for ten minutes.

### Measurement of 7-DHC and cholesterol

After depletion/replenishment, cells were lysed in 1 ml of ice-cold methanol, scraped off the dishes, and sonicated. Two ml of chloroform (containing ^13^C cholesterol as internal standard) were added to 1 ml of cell lysate and vortexed for 10 s. Then, 0.75 ml of KCl 0.7% were added and vortexed for 10 s. The samples were centrifuged at 1000 g during 15 min at 4 °C and chloroform phase (lower) was separated in a glass tube, evaporated using a Speed Vac (Thermo Fisher Scientific, Barcelona, Spain) and resuspended in 200 μl of methanol/chloroform (3:1)^56^. For LC-Q-TOF-based lipid molecular species analyses, lipid extracts were subjected to liquid chromatography-mass-spectrometry using a HPLC 1290 series coupled to ESI-Q-TOF MS/MS 6520 (Agilent Technologies, Barcelona, Spain). Two microliters of lipid extract were injected onto an XBridge BEH C18 shield column (100 mmL × 2.1 mm ID × 1.7 μm; Waters, Milford, MA, USA) kept at 80 °C. The mobile phases, delivered at 0.5 ml/min, consisted of ammonium formate (20 mM at pH 5) (A) and methanol (B). The gradient started at 50% B and reached 70% B in 14 min and was followed by a slow gradient of 70–90% B over 50 min and an isocratic separation at 90% B for 15 min. The mobile phase B subsequently reached 100% over 5 min and was maintained for an additional 5 min^57^. Data were collected in positive electrospray ionization-TOF operated in full-scan mode at 100–3000 m/z in an extended dynamic range (2 GHz) (MassHunter Data Adquisition Sofware, Agilent Technologies, Barcelona, Spain), using N_2_ as nebulizer gas (5 L/min, 300 °C). The capillary voltage was 3500 V with a scan rate of 1 scan/s.

For targeted search of cholesterol, 7-DHC and 13C-cholesterol (internal standard) MassHunter Qualitative Analysis Software (Agilent Technologies, Barcelona, Spain) was used. Specifically, “Find by formula” algorithm was applied to select the main ion for each compound. These ions ((M + H) + (−H2O) = 367.3360 for 7-DHC, (M + H) + (−H2O) = 369.3519 for cholesterol and (M + H) + (−H2O) = 370.3519 for 13C cholesterol) were used for metabolite quantification^56^.

### Data analysis

The Shapiro–Wilk test was used to determine whether variables followed a normal distribution. A Student’s t-test was used to determine differences between normally distributed variables, otherwise a Man–Whitney’s U-test was applied. Differences were considered statistically significant when p < 0.05.

## Additional Information

**How to cite this article**: Gou-Fàbregas, M. *et al*. 7-dehydrocholesterol efficiently supports Ret signaling in a mouse model of Smith-Opitz-Lemli syndrome. *Sci. Rep.* **6**, 28534; doi: 10.1038/srep28534 (2016).

## Supplementary Material

Supplementary Information
