# Supplementary material for: 7-dehydrocholesterol efficiently supports Ret signaling in a mouse model of Smith-Opitz-Lemli syndrome
Source: Sci Rep. 2016 Jun 23;6:28534. doi: 10.1038/srep28534 (PMC4917867; doi:10.1038/srep28534)
Supplement: Supplementary Information [file srep28534-s1.pdf]

SREP-15-32490A

7-dehydrocholesterol efficiently supports Ret signaling in a mouse model of Smith-Optiz-Lemli syndrome

Myriam Gou-Fàbregas[1,2,*], Anna Macià[1], Carlos Anerillas[1], Marta Vaquero[1], Mariona Jové[3], Sanjay Jain[4], Joan Ribera[1], and Mario Encinas[1,*]

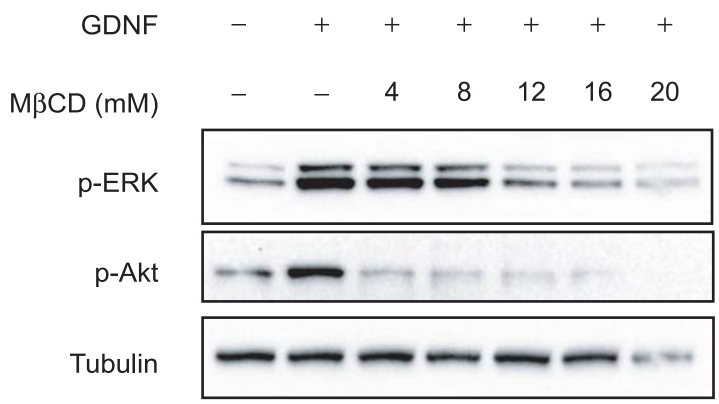

Supplemental Figure 1

Depletion of membrane cholesterol impairs Ret downstream signaling. MG87 cells were treated with methyl-β-cyclodextrin (MβCD) at the indicated concentrations for 30 min to deplete cholesterol from the cell membrane, followed by GDNF stimulation (50 ng/ml, 10 min). Total lysates were resolved by SDS-PAGE and probed with the indicated antibodies.

Supplemental Table 1. List of antibodies used in this study

Antibody	Company	Catalog #	Use	Dilution
Calbindin	Swant	CB38a	Immunofluorescence	1/500
Caveolin-1	BD Transduction	610406	Western Blot	1/1000
c-Src	Upstate	16-182	Western Blot	1/2000
Flotillin-1	BD Transduction	610820	Western Blot	1/1000
Gfrα1	Abcam	ab8026	Western Blot	1/1000
GM-1 (HRP-conjugated cholera toxin B subunit)	Millipore	227041	Western Blot	1/2000
Phospho-Akt	Cell Signalling	4060	Western Blot	1/1000
Phospho-Erk	Cell Signalling	9101	Western Blot	1/10000
PGP9.5	Millipore	AB1761	Immunofluorescence	1/500
TfR	Zymed	13-6800	Western Blot	1/2000
Tubulin	Sigma	T5168	Western Blot	1/50000
